# Alcohol use and its determinants among adults living with HIV/AIDS in Ethiopia: a systematic review and meta-analysis

**DOI:** 10.1186/s12954-021-00503-6

**Published:** 2021-05-17

**Authors:** Birhanie Mekuriaw, Zelalem Belayneh, Alemayehu Molla, Tsegaye Mehare

**Affiliations:** 1 Department of Psychiatry, College of Health and Medical Science, Dilla University, Dilla, Ethiopia; 2 Department of Biomedical Science, College of Health and Medical Science, Dilla University, Dilla, Ethiopia

**Keywords:** Prevalence, Alcohol use, Harmful drinking, Determinants, HIV/AIDS, Ethiopia

## Abstract

**Background:**

Alcohol use is a common practice of almost all communities worldwide and it is more common among persons with HIV infection. Alcohol consumption among people with HIV/AIDS may result in poor treatment adherence, further immunity suppression and increase the risk of comorbid illness (diseases) which collectively diminish the anti-retroviral therapy responses. Although there are separate studies conducted regarding alcohol use among people with HIV/AIDS in Ethiopia, the finding results are highly variable and inconsistent. Therefore, conducting a systematic review and meta-analysis has a paramount importance to show the pooled prevalence of alcohol use and to identify its determinants among people with HIV/AIDS.

**Methods:**

A systematic search of electronic databases of PubMed/Medline, Science Direct, Hinnari and Cochrane library was employed. Additionally, the grey literature was searched from Google and Google Scholar. Data were extracted using a standardized data extraction format prepared in Microsoft Excel . STATA-version 14 statistical software was used for analysis. Heterogeneity of primary studies was found as evaluated using the *I*^2^ test result. As a result, a random-effect meta-analysis model was used to estimate the pooled prevalence of alcohol use.

**Results:**

A total of 22 primary studies which comprises 8,368 study participants were included in this systematic review and meta-analysis. The pooled prevalence of lifetime, current and hazardous alcohol use among HIV patients in Ethiopia were 36.42% [95% CI (19.96, 52.89)], 19.00% [95% CI (12.98, 25.01)] and 21.64% [95% CI (12.72, 30.55)], respectively. Khat chewing [OR = 3.53, (95% CI 1.31, 9.51)] and cigarette smoking [OR = 7.04, (95% CI 3.53, 14.04)] were found as statistically significant determinants of hazardous alcohol use among people with HIV infection.

**Conclusions:**

The result of this review showed that alcohol drinking is highly practiced among people with HIV/AIDS in Ethiopia. The magnitude of alcohol use was highly variable based on the screening methods used to measure alcohol use. Comorbid substance use (khat and cigarette) increases the risk of alcohol consumption among HIV patients. This suggests a need for designing appropriate and culturally applicable intervention programs and policy responses.

*Trial registration* PROSPERO 2019, “CRD42019132524.”

**Supplementary Information:**

The online version contains supplementary material available at 10.1186/s12954-021-00503-6.

## Background

Alcohol is a psychoactive substance having dependency and natural tendency of craving [1]. It has also a toxic effect of different human organs, particularly liver. Nowadays, alcohol use is a major cause of morbidity and mortality worldwide and of immense importance for all health professionals. Alcohol use among people with HIV infection is highly prevalent, and it is a public health problem across the globe [2, 3]. In many societies today, alcoholic beverages are commonly consumed, specifically in developing countries where different homemade and culturally acceptable alcoholic beverages are available [4, 5].

Alcohol is considered as the third leading causes of death that contributes to 3 million deaths each year and responsible for about 5.1% of the overall global burden of disease [6]. The morbidity and mortality rates attributed to alcohol is expected to be greater than the combined numbers of deaths due to human immunodeficiency virus (HIV), acquired immunodeficiency syndrome (AIDS) and tuberculosis globally [7, 8]. Alcohol also affects the development of nations as it accounts for 13.5% of the total death of productive age groups (age 20–39 years) [9].

There is a causal relationship between harmful drinking of alcohol and the incidence of HIV infection in which people who are alcohol users more likely than the general population to contract HIV and vice versa [10, 11]. Studies showed that medication non-adherence, psychological distress, poor quality of life, substance use other than alcohol and poor social support are some of the factors that contribute to the initiation of alcohol use among people living with HIV/AIDS [12, 13]. On the other hand, family history of alcohol or other substance use, poor coping skill to accept their HIV positive sero-status and hazardous alcohol consumption increases the risk of further HIV infection [14, 15].

Despite the high prevalence of alcohol use and its complex medical and psychosocial consequences, addressing alcohol use problems among people with HIV/AIDS is still a challenging task as people usually report by decreasing or totally denying their alcohol intake due to the fear of their social value and violation of medical advices. This may hinder the treatment outcome of people with HIV/AIDS and increases the mortality and comorbidity of other medical and psychosocial disturbances [14, 16].

Alcohol use among people with HIV/AIDS can damage or weaken the already compromised immune system. This intern may fasten the progression of HIV/AIDS by increasing their viral load and vulnerability for other comorbid medical problems (diseases) like hepatitis, respiratory disease, cognitive impairments and physical weakness. Besides, people with alcohol use are more likely to engage different risky behaviors such as unsafe sex, multiple sexual partners and drug abuse which may further increase the risk of HIV transmission [17, 18].

The prevalence of alcohol use among people with HIV/AIDS was reported as it is highly variable in Ethiopia ranging from 6.5 to 32.6% [12, 19]. Despite such variability of results, to the best of ours’ knowledge; there is no published systematic review reporting the pooled prevalence and determinants of alcohol use among people with HIV/AIDS. Therefore, providing concise, comprehensive and summarized results of alcohol use and its associated factors can help for health professional and medical administrators to adapt strategies for the prevention, early identification and intervention of alcohol use among people with HIV/AIDS.

## Methods

### Reporting and protocol registration

This review followed the Preferred Reporting Items for Systematic Reviews and Meta-analysis guideline (PRISMA-P) protocol (Additional file 1). The review protocol has been registered in the International Prospective Register of Systematic Reviews (PROSPERO) with registration number of “CRD42019132524.”

### Databases and search strategies

The systematic search of PubMed/Medline, Science direct, Hinari and Cochrane library databases was done following comprehensive search strategies. Key terms used to search kinds of literature from PubMed were “Prevalence” OR “Magnitude” OR “Epidemiology” AND “Alcohol use Disorders” OR “Alcohol use” OR “Alcohol consumption” OR “Problematic Alcohol use” OR “Alcohol abuse” OR “Alcohol dependence” combining with “HIV patients” OR “Human immunodeficiency virus,” “HIV infection,” OR “People with HIV” OR “AIDS patients,” AND “Determinants” OR “Associated factors” OR “Risk factors” AND “Ethiopia.” Moreover, we searched gray literature from Google and Google Scholar. For studies whose full text was not accessible, we contacted the first authors via email and request for the full text of the paper.

### Inclusion criteria

Observational studies (cross-sectional, case–control and cohort studies) reporting the prevalence of alcohol use and/or factors associated with alcohol use among patients with HIV/AIDS in Ethiopia were considered to be included in this review. Primary studies which assured the following criteria were considered as eligible for this systematic review and meta-analysis.

*Population* Studies conducted among HIV infected adults (age ≥ 18 year) in Ethiopia were eligible to be included in this review.

*Measurements *Studies reporting the prevalence of alcohol use using standardized measurement tool or questionnaires such as AUDIT (Alcohol Use Disorder Identification Test), CAGE (Cut down, Annoyed, Guilty feeling and Eye opener) or other measurement tools were included.

*Language* Primary studies published in English or having additional English version were included.

*Publication condition and article type* Both published and unpublished research articles were included, but conference papers and qualitative studies were not eligible.

*Publication year* Primary studies published until August 2019 were included in this review and meta-analysis.

### Selection of studies

In the first stage, titles and abstracts of primary studies were stored and managed in EndNote reference system version 7. Then, duplicated studies were concisely removed from the EndNote. Two of the authors (BM and ZB) were responsible for reviewing titles and abstracts of all studies independently, and any disagreements between the two assessors were solved through discussion and reached to consensuses. After title and abstract assessment, articles identified as relevant were considered for further evaluation by a thorough reading of their full texts. Articles fulfilling the minimum eligibility criteria during their full text evaluation were included in this systematic review and meta-analysis.

### Data extraction

The two authors (BM and AM) extracted all necessary data, independently using a standardized data extraction format prepared in Microsoft Excel. For the first outcome (prevalence of alcohol use), the data extraction format was prepared with different columns including first author’s name, publication year, region of the study conducted, alcohol use measurement techniques, sample size and prevalence of alcohol use. For the second outcome (determinants of alcohol use), data were extracted using two by two tables for each predictor. In this review, variables considered as correlates of alcohol use by at least two primary studies were included in this meta-analysis to show the pooled odds ratio (effect size) on hazardous alcohol use. Any disagreement during data extraction was solved through discussion.

### Definition of terms

*Life time alcohol use* In this study, it refers to when studies assessed and reported the magnitude of alcohol consumption as use of any amount of alcohol in the participant’s life time.

*Current alcohol use* This refers when the studies reported consumption of alcohol in any amount in the past three to/or twelve months.

*Hazardous alcohol use* It was considered when studies assessed and reported the magnitude of alcohol consumption according to standardized measurement tools used to estimate the level of problematic alcohol consumption such as Alcohol Use Disorder Identification Test (AUDIT).

### Quality assessment

Two authors (AM and TM) independently assessed the quality of each primary study using Newcastle–Ottawa Quality Assessment tool adapted for cross-sectional and case–control studies [20, 21]. These tools have different indicators with three main sections used to evaluate the methodological quality, comparability and overall quality of the original articles, independently. The quality assessment tool has comprised of 10 scores indicating different quality levels of papers (1/ “high quality” if papers have a total score of greater than/equal to 6 and 2/ “low quality” when papers scored less than 6 points). Accordingly, studies with a high quality assessment level were included in this review and meta-analysis.

### Measurements of outcomes

Our systematic review and meta-analysis has two main objectives. The first objective was to determine the pooled prevalence of alcohol use (current use, life time use and hazardous drinking) among HIV patients in Ethiopia. The pooled prevalence of alcohol use was obtained by dividing the total number of alcohol users to the total number of samples and multiplied by hundred (100). The second objective was to identify the pooled effect of determinants for alcohol use. The odds ratio was calculated from primary studies using two by two tables to identify determinants of alcohol use (khat chewing and cigarette smoking) (Additional file 2).

### Data analysis

The extracted data were imported to STATA Version 14.0 (software) for analysis. Characteristics of original articles were described using a table and a forest plot. The standard error of prevalence for each original article was calculated using the binomial distribution formula.

Heterogeneity among primary studies was checked by using heterogeneity χ^2^ test and *I*^2^ test [22]. The heterogeneity tests result indicated that there was a significant heterogeneity between primary studies. Therefore, a random-effect meta-analysis model was used to estimate the Der Simonian and Laird’s pooled prevalence alcohol use and its determinants. In addition, a subgroup analysis was done based on study location and sample size to minimize the random variations between the point estimates of primary studies.

Publication bias was also examined by performing Egger’s correlation test and symmetrical distribution of funnel plot [23, 24]. The results of Egger’s tests indicated that there was a publication bias in the current and hazardous level of alcohol consumption as evidenced by *P* < 0.001 and *P* = 0.013, respectively. This publication bias was addressed by conducting leave-one-out analysis.

## Results

### Identification and selections of studies

**Fig. 1:**
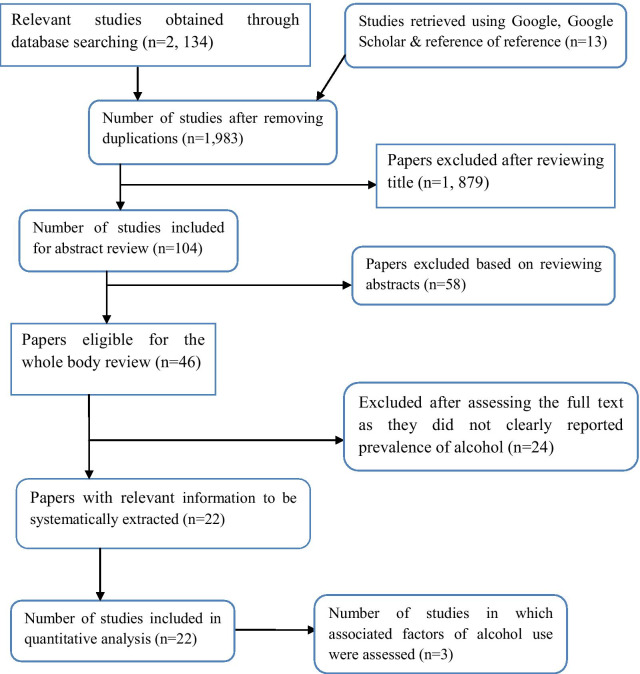
Diagramatic presentation of the selection process of articles included in the systematic review and meta-analysis

In the first step of our search, a total of 2147 articles were retrieved using PubMed/Medline, Science direct, Hinari, Cochrane library, Google and Google Scholar. Of these articles, 164 articles were removed due to duplication and other 1937 articles were excluded after their title and abstract evaluation. The remaining 46 primary studies were considered for further eligibility assessment through careful reading of their full texts. After their full text evaluation, 24 articles were further excluded due to differences in the study population, study settings and outcome interests. Finally, 22 articles were found to be eligible and included in the systematic review and meta-analysis (Fig. 1).

### Characteristics of included articles

Table 1 shows summary of the characteristics of 22 primary studies included in our systematic review and meta-analysis. Regarding study location of primary studies, five from Amhara region [25–29], seven from Oromia [12, 13, 30–34], three from Addis Ababa [19, 35, 36], other three from SNNPR [37–39], two from Tigray [40, 41], one from Dire Dawa [42] and the other one from Hararie [43]. The publication year of all included articles range from 2005 to 2019. In most primary studies, self-report was used to measure the current and lifetime alcohol use whereas five primary studies used alcohol use disorder identification test (AUDIT) to measure level of alcohol consumption among people with HIV/AIDS (Table 1).

**Table 1:** Summary table of the prevalence of alcohol use (based on screening techniques) among people with HIV/AIDS in Ethiopia for 22 primary studies included in this systematic review and meta-analysis

Region	Study site	Author	Publication year	Sample size	Alcohol use measurement	Quality score	Lifetime prevalence of alcohol use (95% CI)	Current prevalence of alcohol use (95% CI)	Hazardous alcohol use (95% CI)
Amhara	Bahir-Dare	Wondemagegn et al. [25]	2013	269	Self-report	6.5	35.30 (29.59, 41.01)	–	–
	Gondar	Azagew et al. [28]	2017	422	Self-report	7	–	10.20 (7.31, 13.09)	–
	Wello	Abaynew et al. [29]	2011	320	Self-report	7	36.20 (30.93, 41.47)	–	–
	Debark	Bitew H et al. [27]	2016	393	Self-report	6	77.10 (72.95, 81.25)	38.40 (33.59, 43.21)	–
	N. Shewa	Basha A et al. [26]	2019	422	Self-report	7	–	29.60 (25.24, 33.96)	–
Addis Ababa	Addis Ababa	Gebremariam et al. [19]	2017	417	Self-report	7	6.50 (4.13, 8.87)	–	–
	Addis Ababa	Seme A et al. [35]	2005	207	Self-report	6	55.10 (48.32, 61.88)	–	–
	Addis Ababa	Dessie et al. [36]	2011	601	Self-report	7.5	–	23.30 (19.92, 26.38)	–
Dire Dawa	Dire Dawa	Lifson et al. [42]	2017	322	Self-report	7	–	43.00 (37.59, 48.41)	–
Oromia	Jimma	Soboka et al. [12]	2014	389	AUDIT	8	–	–	32.60 (27.94, 37.26)
	Bishoftu	Bultum JA et al. [13]	2018	527	AUDIT	9	–	–	14.20 (11.22, 17.18)
	Jimma	Yitbarek et al. [30]	2019	328	Self-report	7	13.70 (9.98, 17.42)	4.30 (2.10, 6.50)	–
	Assela	Segni MT et al. [32]	2017	418	Self-report	6	–	13.60 (10.31, 16.89)	–
	Jimma	Bosho et al. [33]	2018	268	Self-report	7	38.10 (32, 29, 43.91)	14.20 (10.02, 18.82)	–
	Jimma	Deribe et al. [31]	2008	343	Self-report	6	35.30 (30.34, 40.36)		–
	Hararge	Dedha et al. [34]	2017	437	Self-report	7	–	18.30(14.67, 21.93)	–
Harerie	Harar	Motumma et al. [43]	2019	420	Self-report	8	–	16.60 (12.86, 19.94)	–
SNNPR	Hawassa	Duko et al. [39]	2019	195	AUDIT	9	–	–	31.80 (25.26, 38.34)
	Gedeo	Belayneh et al. [37]	2019	412	AUDIT	8	–	–	22.10 (18.09, 26.11)
	Gamo	Animut M et al. [38]	2019	684	AUDIT	7	–	–	8.80 (6.69, 10.92)
Tigray	Mekelle	Tilahun B et al. [40]	2017	234	Self-report	7.5	–	6.00 (2.96, 9.04)	–
	Central zone	Woldehawaria et al. [41]	2017	240	Self-report	6	30.90 (25.99, 35.81)	12.60 (9.07, 16.13)	–
Overall prevalence							36.42 (19.96, 52.89)	19.00 (12.99, 25.01)	21.64 (12.72, 30.55)

### Meta-analysis

#### Prevalence of lifetime alcohol use

**Fig. 2:**
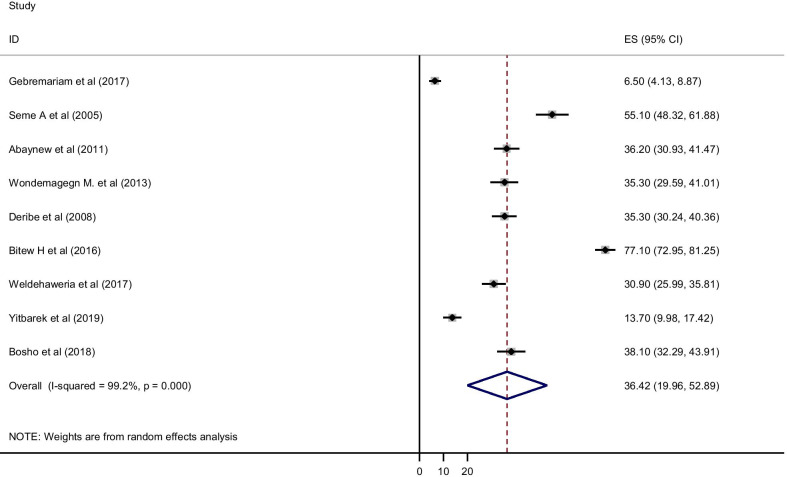
Forest plot for the pooled prevalence of lifetime alcohol use among HIV/AIDS patients in Ethiopia, 2019

From a total of 22 studies, nine of them reported the lifetime prevalence of alcohol use. Accordingly, the pooled prevalence of lifetime alcohol use among HIV/AIDS patients in Ethiopia was 36.42% [95% CI (19.96, 52.89)]. Heterogeneity was observed across the studies which is uncovered by *I*^2^ statistic (*I*^2^ = 99.2%, *p* value < 0.001). Therefore, a random-effect model was conducted to estimate the pooled prevalence of alcohol consumptions. Publication bias was also checked using Eggers's tests and publication bias was not observed as evidenced by (*P* = 0.06) (Fig. 2).

#### Prevalence of current alcohol use

**Fig. 3:**
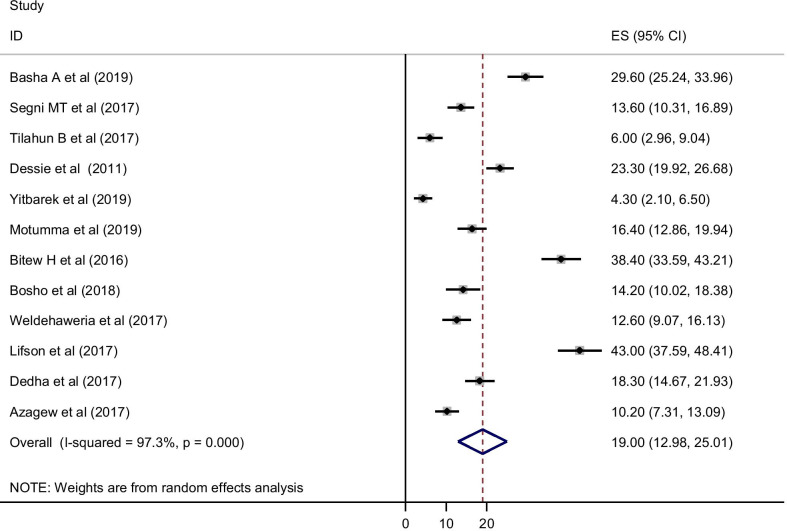
Forest plot for the pooled prevalence of current alcohol use among HIV/AIDS patients in Ethiopia, 2019

Among 22 studies, 12 studies reported the prevalence of current alcohol use among HIV/AIDS patients in Ethiopia. The pooled prevalence of current alcohol use was 19.00% [95% CI (12.98, 25.01)]. *I*^2^ test showed that a significant heterogeneity (*I*^2^ = 97.3% and *P* < 0.001) and publication bias was noticed using eggers test (*P* < 0.001) (Fig. 3).

#### Prevalence of hazardous alcohol use

**Fig. 4:**
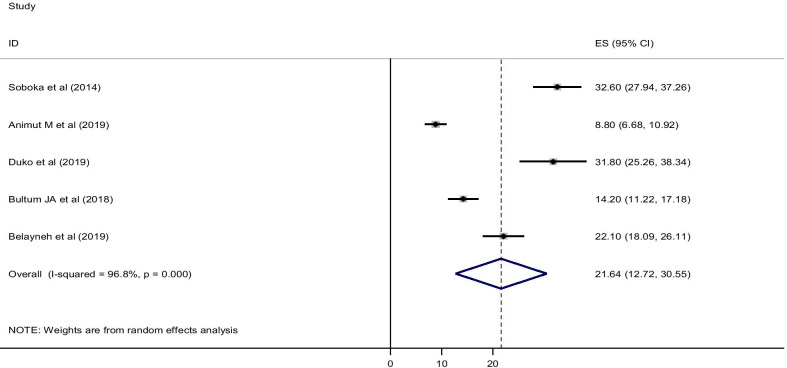
Forest plot for the pooled prevalence of hazardous alcohol use among HIV/AIDS patients in Ethiopia, 2019

Five primary studies reported the magnitude of hazardous alcohol consumption, and the pooled prevalence of hazardous alcohol use was found to be 21.64% [95% CI (12.72, 30.55)]. Significant heterogeneity was observed across studies as evidenced by (*I*^2^ = 96.8% and *P* < 0.001). The result of eggers test was *P* = 0.013 which signifies the presence of publication bias (Fig. 4).

### Subgroup analysis

The level of alcohol consumption is determined by culture, attitude and socio-demographic characteristics, availability of alcohol and individual characteristics (age, sex, marital status and educational level) [44]. In Ethiopia, there are different homemade traditional alcoholic beverages commonly consumed at different parties and ceremonies. However, the accessibility and cultural acceptability of such alcoholic beverages vary from region to region. For example, alcohol is a culturally acceptable and drinking is commonly practiced in the Northern part of the country where as alcohol drinking is considered as a social taboo in some eastern and southern part of Ethiopia. To minimize random variations between the point estimates of primary studies, we conducted subgroup analysis based on the region where primary studies have been conducted. Accordingly, the higher prevalence of lifetime, hazardous and current alcohol use was found among studies conducted at Amhara region, SNNPR and eastern part of the country, respectively. Regarding sample size, higher prevalence of alcohol consumption was found among studies which have a sample size of greater than 340 both in current and lifetime alcohol use (Table 2).

**Table 2:** Subgroup analysis results of alcohol use among people living with HIV/AID in Ethiopia

Types of alcohol use	Region	Number of studies	Prevalence (95% CI)	*P* value	*I*^2^
Lifetime use	Addis Ababa	2	30.69 (− 16.93, 78.32)	*P* < 0.001	99.4%
	Amhara	3	49.58 (20.42, 78.74)	*P* < 0.001	99.0%
	Oromia	3	28.93 (12.42, 45.43)	*P* < 0.001	97.2%
	Tigray	1	30.90 (25.99, 35.81)		
Hazardous	SNNPR	3	23.31 (5.28, 41.34)	*P* < 0.001	97.0%
	Oromia	2	20.62 (7.64, 33.60)	*P* < 0.001	97.6%
Current use	Amhara	3	25.99 (8.36, 43.61)	*P* < 0.001	98.3%
	Oromia	4	12.50 (5.66, 19.34)	*P* < 0.001	94.5%
	Tigray	2	9.42 (2.77, 15.70)	*P* = 0.005	87.0%
	*Others	2	29.62 (3.55, 55.69)	*P* < 0.001	98.5%
	Addis Ababa	1	23.30 (19.92, 26.68)		

Types of alcohol use	Sample size	Number of studies	Prevalence (95% CI)	*P* value	*I*^2^
Current use	≥ 340	8	20.17 (14.29, 26.04)	*P* < 0.001	95.3%
	< 340	4	16.67 (3.56, 27.79)	*P* < 0.001	98.3%
Lifetime use	≥ 340	4	37.43 (4.30, 70.57)	*P* < 0.001	99.7%
	< 340	5	35.54 (21.46, 49.63)	*P* < 0.001	97.2%

^*^Others = Dire Dawa and Harerie (eastern part of the country)

### Publication bias and sensitivity analysis

In this study, publication bias was observed in current and hazardous alcohol consumption which is evidenced by Egger’s test (*P* < 0.001) and (*P* = 0.013), respectively, as well as asymmetrical distributions of studies at funnel plot structure. Sensitivity analysis was performed after removing three primary studies for current alcohol use and one study for hazardous alcohol consumption. Thus, the pooled prevalence of current and hazardous alcohol consumption remains similar with the previous pooled prevalence. This indicants that the review is stable and valuable.

### Determinants of hazardous alcohol use

**Fig. 5:**
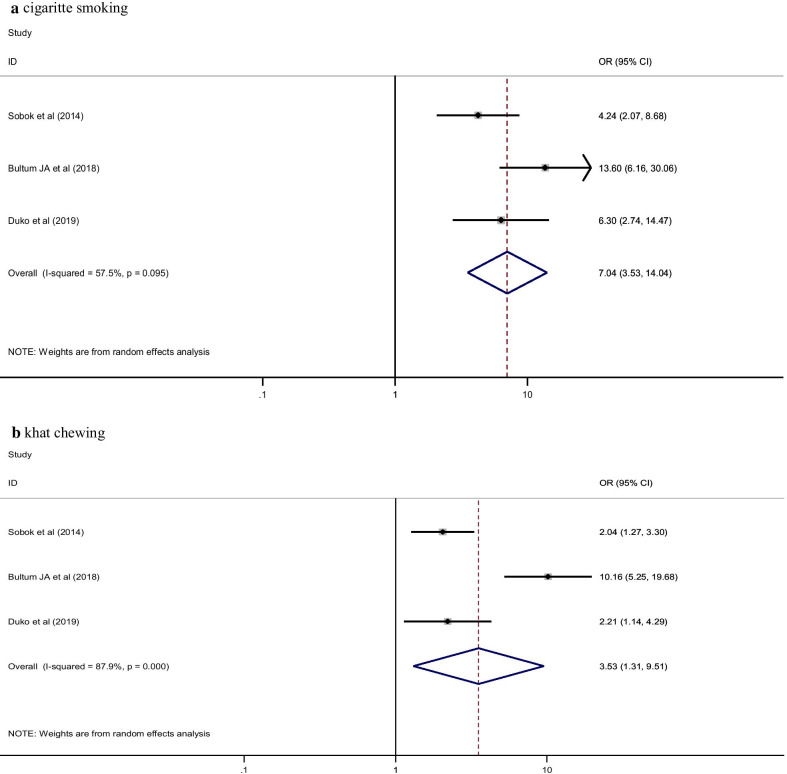
Determinants of hazardous alcohol use among HIV/AIDS patient in Ethiopia, 2019

Regarding determinants of alcohol use, important data from primary studies were available only for hazardous alcohol consumption. Accordingly, khat chewing [OR = 3.53 (95% CI 1.31, 9.51)] and cigarette smoking [OR = 7.04 (95% CI 3.53, 14.04)] were factors associated with hazardous alcohol consumption (Fig. 5).

## Discussion

To the best our knowledge, this systematic review and meta-analysis is the first to determine the pooled prevalence of alcohol use and to identify its determinants among HIV/AIDS in Ethiopia. According to this review, the pooled prevalence of lifetime, current and hazardous alcohol use among HIV patients in Ethiopia were 36.42% [95% CI (19.96, 52.89)], 19.00% [95% CI (12.98, 25.01)] and 21.64% [95% CI (12.72, 30.55)], respectively. The result of this review showed that the pooled prevalence of current and hazardous alcohol consumption was in line with a systematic review and meta-analysis among non-HIV adults in Eastern Africa. However, the lifetime prevalence of alcohol consumption in our review was lower than the East African finding (52%) [45].

The possible reason for the disparity of result reports might be explained by the differences in the sociocultural context of study populations of primary studies included in East African review and our study, i.e., the review done in East Africa was conducted among younger adults. Thus, younger adults are more prone to engage to risky behaviors including alcohol drinking [46]. This could be the reason which increase the prevalence of alcohol use in the East African review among younger adults. Moreover, differences in inclusion and exclusion criteria as well as quality of primary studies might play a role for the discrepancy of these review findings.

The finding of the current systematic review showed the pooled prevalence of life time alcohol use was lower than findings reported from studies conducted among none-HIV/AIDS population in Ethiopia (44.16%) [47]. Difference in study population might be a reason for the discrepancy of the results. However, the pooled prevalence of hazardous alcohol consumption of our finding was high as compared to non HIV population in Ethiopia (8.94%). The possible explanation for this difference might be due to the fact that people with HIV/AIDS often use alcohol as a self-medication to induce sleep, to forget severe pain and as a means of social engagement with other drinkers [13]. In addition, people with HIV/AIDS are more likely to have psycho-social or emotional distresses that might push them to initiate alcohol for the sake of getting episodic relief [48]. On the other hand, the magnitude of hazardous alcohol consumption was comparable with the finding of other systematic review (29.80%) and more similar with the prevalence found in developing country (24.52%) [49].

The subgroup analysis of this review showed a higher prevalence of current, lifetime and hazardous alcohol consumption among studies conducted in the eastern part of the country (29.62%), Amhara region (48.58%) and Oromia region (23.31%), respectively. This indicates that the consumptions of alcohol were variable across regions of the country. The possible explanation for such regional differences on the magnitude of alcohol consumption might be explained by the fact that there is a great difference in the acceptability and accessibility of alcoholic beverages across different regions of Ethiopia. For example, alcohol is considered as a highly acceptable drink, and drinking is commonly practiced during family meal, parties and ceremonies in Northern part of Ethiopia, particularly Amhara region. As a result, there are different homemade alcoholic beverages (Tella, Tej, Areki…) with different and undetermined alcoholic contents prepared every household [5]. These homemade alcoholic beverages are easily available and accessible with low cost. However, most people in Eastern and some southern part of Ethiopia are Islam and protestant religion followers in which alcohol is totally prohibited, and drinking is considered as a social taboo and culturally sanctioned. Therefore, the government and policy makers should give due attention regarding the prevention, early identification and interventions of hazardous alcohol consumption among high risk regions. Despite appropriate measures are implemented currently such as restricting beer advertising and increasing taxes, additional price techniques or solutions like banning below cost selling or volume discount of easily available and accessible homemade alcoholic beverages could be essential to reduce the social and health costs of harmful alcohol use in which further it can increase the life expectancy or save many lives of people with HIV. Advocating and endorsing cultural interventions such as elders and religious father teaching or guidance of alcohol consumption to their religious fellows which are commonly practiced in the eastern and some southern part of the country can be important in collaboration with key stake holders and decision makers.

In this systematic review and meta-analysis, determinants of hazardous alcohol use were identified. Accordingly, comorbid substance use (khat and cigarette) was a predictor of hazardous alcohol use among HIV/AIDS patients in Ethiopia. People with HIV/AIDS having khat chewing behavior were 3.5 times more likely to have hazardous alcohol use as compared to their counterpart. This might be due to the substantial stimulant nature of khat by activating the dopamine activity of the brain [50]. As a result, people commonly use alcohol as a self-medication to calm down their hyperactivity after finishing their khat chewing. Furthermore, people having a habit of chewing are more likely to combine other additional drugs including alcohol [51].

This study also revealed that the odds of hazardous alcohol use among cigarette smokers were increased by 7 times as compared to non-smokers. Psychoactive substances like cigarettes might be used as an antidote so as to alleviate the depressant effect of alcohol as smoking has a stimulant effect that makes smokers to be more active and cheerful [50]. In general, substance use by nature has a kind of interrelation behaviors in which individuals who abuse a single psychoactive substance is more likely to initiate other substances as a result of the dependency and tolerance effects. With this recognition, providing pragmatic service to screen out substance misuse at HIV health care setting will have a paramount importance to treat patients early and prevent further complications.

## Conclusions

The current systematic review and meta-analysis showed that the pooled prevalence of alcohol use among people with HIV/AID was high. Comorbid substance use (khat and cigarette smoking) was found to have statistically significant association with hazardous alcohol consumption. This sounds a need to design and adopt applicable strategies to integrate the prevention, early identification and treatment of alcohol use problems to the HIV/AIDS care services in which it can be helpful to reduce the social and healthcare costs and increase functioning in all area of wellness.

## Limitations of the study

The possible publication bias of primary studies may be the limitation of this systematic review and meta-analysis. In addition, selection of only articles those were written or translated into English might be also a limitation for this study.

## Supplementary Information

**Additional file 1.** PRISMA checklists.

**Additional file 2.** Sample data extraction format.

## Data Availability

All data included in this manuscript are available and can be accessed from the corresponding author.

## Abbreviations

AOR: Adjusted odds ratio
AUDIT: Alcohol use disorder identification test
CI: Confidence interval
HIV/AIDS: Human immunodeficiency virus/acquired immunodeficiency syndrome
PRISMA-P: Preferred reporting items for systematic reviews and meta-analysis
